# IMP-27, a Unique Metallo-β-Lactamase Identified in Geographically Distinct Isolates of Proteus mirabilis

**DOI:** 10.1128/AAC.02945-15

**Published:** 2016-09-23

**Authors:** Nyssa Dixon, Randal C. Fowler, A. Yoshizumi, Tsukasa Horiyama, Y. Ishii, Lucas Harrison, Chelsie N. Geyer, Ellen Smith Moland, Kenneth Thomson, Nancy D. Hanson

**Affiliations:** a Creighton University School of Medicine, Department of Medical Microbiology and Immunology, Center for Research in Anti-Infectives and Biotechnology, Omaha, Nebraska, USA; b Department of Pathology and Microbiology, University of Nebraska Medical Center, Omaha, Nebraska, USA; c School of Medical Technology, Gumma Paz College, Takasakishi, Gumma, Japan; d Shionogi & Co., Ltd., Osaka, Japan; e Toho University School of Medicine, Department of Microbiology and Infectious Diseases, Tokyo, Japan; f CHI Health, Omaha, Nebraska, USA; g University of Louisville School of Medicine, Louisville, Kentucky, USA

## Abstract

A novel metallo-β-lactamase gene, *bla*_IMP-27_, was identified in unrelated Proteus mirabilis isolates from two geographically distinct locations in the United States. Both isolates harbor *bla*_IMP-27_ as part of the first gene cassette in a class 2 integron. Antimicrobial susceptibility testing indicated susceptibility to aztreonam, piperacillin-tazobactam, and ceftazidime but resistance to ertapenem. However, hydrolysis assays indicated that ceftazidime was a substrate for IMP-27.

## TEXT

Metallo-β-lactamases (MBLs) are class B carbapenemases that hydrolyze most β-lactam antibiotics except aztreonam (1, 2, 3). Clinically relevant MBLs include IMP, VIM, GIM, SPM, SIM, KHM, AIM, and NDM family members (4, 5). The majority of IMP and VIM MBLs are found in isolates of Pseudomonas aeruginosa and, in some cases, Klebsiella pneumoniae and Escherichia coli (2, 5). The *bla*_IMP_ and *bla*_VIM_ genes have been discovered in isolates collected from Asia, Europe, Australia, and North and South America (2, 3, 5). One contribution to the spread of MBLs is the mobilization of the genes via integrons (5, 6). Most genes encoding IMP and VIM MBLs are found as gene cassettes in either class 1 or class 3 integrons (3, 4, 5). Here, we report a novel IMP MBL, IMP-27, that was found in a class 2 integron and isolated from two geographically distinct isolates of Proteus mirabilis from the United States. These isolates were collected in 2009 and 2015 from two different patients who were from two different states in the upper plains region of the United States. Pulsed-field gel electrophoresis (PFGE) and plasmid analysis showed that the two isolates were not highly related. NotI restriction analysis demonstrated a ≥8-band difference in the PFGE patterns between the isolates (7), as the isolate from 2009 contained no plasmids and the 2015 isolate contained 4 plasmids. Southern analysis using an 80-bp digoxigenin (DIG)-labeled probe specific for *bla*_IMP-27_ demonstrated that the 2009 isolate housed *bla*_IMP-27_ on the chromosome and the 2015 isolate housed the gene on both the chromosome and the high-molecular-weight plasmids (8). Antimicrobial susceptibilities were determined by a combination of Vitek 2 (bioMérieux, Hazelwood, MO), microbroth panel (Trek Diagnostic Systems, Cleveland, OH), and disk diffusion (9). Results were interpreted using Clinical and Laboratory Standards Institute guidelines (10). Both isolates were resistant to ceftriaxone (MIC, 64 μg/ml) and had MICs of >8 μg/ml to imipenem, meropenem, and doripenem. As expected, the isolates were susceptible to aztreonam by disk diffusion (≥21 mm) but atypically susceptible to ceftazidime (≤4 μg/ml) and piperacillin-tazobactam (≤4 μg/ml).

Multiplex PCR using the ARM-D for β-lactamase ID kit (Streck, Inc.) identified a *bla*_IMP-1_-like gene in both isolates. Flanking primers were used to amplify the entire gene from the two isolates, and sequence analyses of these amplicons identified *bla*_IMP-27_ (GenBank accession number JF894248). The translated product of *bla*_IMP-27_, IMP-27, had 87.4% identity to IMP-8 and 79.7% identity to IMP-1 (11). The isoelectric point for IMP-27 was determined to be 6.4 (12). To determine the kinetic parameters of IMP-27, *bla*_IMP-27_ was PCR amplified from total DNA extracted from the 2009 P. mirabilis isolate (13). The PCR product was subcloned into a pET-9a-positive (pET-9a^+^) expression vector (Novagen, Darmstadt, Germany) and overexpressed in E. coli BL21(DE3)/pLysS (Promega, Madison, WI) using 1 mM isopropyl-β-d-thiogalactopyranoside (IPTG). IMP-27 was purified by anion-exchange chromatography, HiTrap Q FF column (GE Healthcare Life Sciences, Little Chalfont, United Kingdom), and hydrophobic interaction chromatography using the HiTrap Butyl HP (GE Healthcare Life Sciences). The purity of the final preparation was >98% as determined by SDS-PAGE. The kinetic properties of the purified IMP-27 (Table 1) were calculated for imipenem, meropenem, cefotaxime, ceftazidime, cefepime, aztreonam, piperacillin, and nitrocefin by measuring the initial hydrolysis rates using a UV-2550 spectrophotometer (Shimadzu Co., Kyoto, Japan). *K_i_* values for EDTA and dipicolinic acid were determined using nitrocefin as the reporter substrate. The hydrolytic efficiencies of IMP-27 were experimentally compared to IMP-1. IMP-27 hydrolyzed all β-lactams tested with the exception of aztreonam and piperacillin. *K_i_* values of IMP-27 for aztreonam and piperacillin were >23,000 μM and 3,000 μM, respectively, whereas IMP-1 showed a high affinity against piperacillin with a *K_m_* value of 330 μM. The hydrolytic efficiency (*k*_cat_/*K_m_*) of imipenem for IMP-27 was 1.1 × 10^5^ M^−1^ s^−1^ compared to 1.8 × 10^6^ M^−1^ s^−1^ for IMP-1. IMP-27 and IMP-1 were efficiently inactivated by metal chelators showing *K_i_* values for EDTA of 11 ± 0.9 mM and 1.5 ± 0.11 mM and dipicolinic acid *K_i_* values of 4.6 ± 0.13 mM and 0.44 μM ± 0.017 mM, respectively. The combination of amino acid substitutions observed in IMP-27 resulted in a lower catalytic efficiency compared to IMP-1 for imipenem. In addition, the *K_i_* values of EDTA and dipicolinic acid for IMP-27 were ∼10 times higher than those observed for IMP-1.

**TABLE 1:** Kinetic parameters of purified IMP-27 and IMP-1

Antibiotic	IMP-27			IMP-1		
	*K_m_* or *K_i_* (μM)	*k*_cat_ (s^−1^)	*k*_cat_*/K_m_* (M^−1^ s^−1^)	*K_m_* or *K_i_* (μM)	*k*_cat_ (s^−1^)	*k*_cat_*/K_m_* (M^−1^ s^−1^)
Piperacillin	3,000 ± 390^*a*^	ND^*b*^	ND	330 ± 12^*a*^	40 ± 1.5^*c*^	1.2 ×10^5^
Cefotaxime	24 ± 1.9	20 ± 0.66	8.6 × 10^5^	9.8 ± 1.2	16 ± 0.34	1.6 ×10^6^
Ceftazidime	54 ± 4.3	0.7 ± 0.03	1.3 × 10^4^	46 ± 3.8	7.4 ± 0.10	1.6 ×10^5^
Cefepime	53 ± 3.6	8.1 ± 0.37	1.5 × 10^5^	42 ± 1.0	15 ± 0.36	3.6 × 10^5^
Aztreonam	>23,000 NH^*d*^	NH	ND	>23,000 NH	NH	ND
Imipenem	310 ± 19^*a*^	34 ± 0.38	1.1 × 10^5^	28 ± 2.0	52 ± 1.5	1.8 × 10^6^
Meropenem	2.3 ± 0.8^*a*^	3.4 ± 0.53	1.5 × 10^6^	4.3 ± 0.7	8.2 ± 0.71	2.0 ± 10^6^
Nitrocefin	36 ± 3.1	370 ± 15	1.0 × 10^7^	3.9 ± 0.37	270 ± 17	7.0 ×10^7^

a The *K_m_* values were measured as *K_i_* with nitrocefin as the reporter substrate.

b ND, not determined.

c The *k*_cat_ values were derived from initial rate measurements at more than 5 times higher than the concentration of *K_i_*.

d NH, no hydrolysis detected.

**FIG 1:**
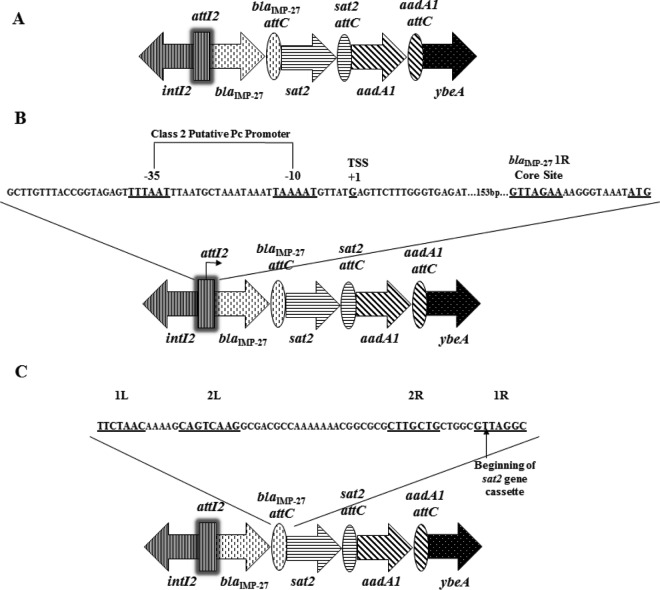
(A) Schematic representation of the class 2 integron harbored within the P. mirabalis isolates. The vertical rectangle represents the *attL2* site found in the *intL2* gene cassette. Each gene cassette consists of a gene, represented by an arrow, and its corresponding *attC* site, represented by ovals. (B) Schematic representation of the class 2 integron putative promoter sequence, transcriptional start site, core site, and *bla*_IMP-27_ start codon. (C) Schematic representation of the P. mirabilis integron 2 *dfrA1 attC* recombination site.

To determine the genetic backbone of *bla*_IMP-27_ in these isolates, a series of PCR amplicons was generated using the GenomeWalker universal kit (Clontech, Mountain View, CA) with primers listed in Table 2. Sequence analysis of these PCR fragments identified the *bla*_IMP-27_ gene within a class 2 integron (Fig. 1A) (GenBank accession number KF501391). *bla*_IMP-27_ was identified within the first gene cassette instead of the dihydrofolate reductase (*dfrA1*) gene, which typically occupies this position (14, 15, 16). It has been shown experimentally that gene cassettes located immediately after the integron promoter have increased expression compared to subsequent gene cassettes (17, 18, 19). The putative class 2 integron −35 and −10 promoter sequences, the transcriptional start site (TSS), the core site, and the *bla*_IMP-27_ start codon, all of which are required for gene cassette transcription and site-specific recombination, are represented within the *attL2* recombination site (Fig. 1B) (20, 21, 22).

**TABLE 2:** Primers utilized in combination with GenomeWalker to determine the genetic backbone of *bla*_IMP-27_

Primer name	Primer type	Sequence (5′ to 3′)	Target	Source
IMP2813F	Primary	CGAGAAGCTTGAAGAAGGT	3′ *bla*_IMP_	This work
AP1		GTAATACGACTCACTATAGGGC		GenomeWalker
IMP-PMF2	Nested	CAAGACAACGTAGTAGTTTGG	3′ *bla*_IMP_	This work
AP2		ACTATAGGGCACGCGTGGT		GenomeWalker
IMP-PMR1	Primary	GTATCTTTAGCAGTAAATGG	5′ *bla*_IMP_	This work
AP1		GTAATACGACTCACTATAGGGC		GenomeWalker
IMP-PMR5	Nested	CCACCAAACGTGTTTAGTAAC	5′ *bla*_IMP_	This work
AP2		ACTATAGGGCACGCGTGGT		GenomeWalker
PmintI2F1	Primary	CCTGACCTCTTCACTGCCC	3′ *intI2*	This work
AP1		GTAATACGACTCACTATAGGGC		GenomeWalker
PmintI2F2	Nested	CAGCAGACATGTAGCCATAAACACGC	3′ *intI2*	This work
AP2		ACTATAGGGCACGCGTGGT		GenomeWalker

A cassette typically consists of a resistance gene linked to an *attC* site (23, 24, 25, 26). Each gene cassette’s *attC* site contains four core sites: 1L, 2L, 2R, and 1R. Core site 1L is typically the reverse complement of 1R and 2L is typically the reverse complement of 2R, resulting in the formation of a hairpin structure upon recombination. Within the 1R core site is a conserved GTT sequence, which is required for the binding of the IntI2 enzyme and thus recombination of a gene cassette between the G and T bases (22, 27, 28, 29). Within the *bla*_IMP-27_ *attC* site, this recombination site lies directly adjacent to the beginning of the *sat2* gene cassette (Fig. 1C).

To our knowledge, the data presented in this report describe several firsts. (i) This is the first publication identifying an IMP-type MBL in a P. mirabilis isolate. (ii) We report the genetic identification and kinetic analysis of a novel IMP MBL, IMP-27. The kinetic data support the susceptibility to both aztreonam and piperacillin. It is interesting that this enzyme did not confer resistance to ceftazidime for the P. mirabilis isolates even though the kinetic data indicated ceftazidime hydrolysis. (iii) *bla*_IMP-27_ was identified in a class 2 integron, which is very unusual, as class 2 integrons contain a premature stop codon in their integrase gene, leading to little genetic diversity among class 2 integrons (16, 19, 20, 21). (iv) This novel IMP MBL is distinct from most other IMP β-lactamases and has been identified in three geographically distinct locations within a 6-year period. Two of the unrelated isolates were described in this report, and the other P. mirabilis isolate was identified in Ontario, Canada (30).

The identification of MBLs in P. mirabilis is rare and may be due to the unique susceptibility profile of these isolates. Organisms expressing only a MBL are typically resistant to all β-lactams except aztreonam, but P. mirabilis isolates producing IMP-27 are also susceptible to ceftazidime and piperacillin-tazobactam; therefore, the identification of this novel MBL found in three geographically distinct P. mirabilis isolates is a concern. It is important for clinical microbiologists to be aware of this unique susceptibility profile when this MBL is harbored within P. mirabilis. Identification of this unique susceptibility profile by clinical microbiologists will aid in the surveillance and infection control measures needed to curb the spread of these types of resistance genes. It may also be prudent to closely monitor patients infected with an IMP-27-producing organism for the emergence of resistance if either ceftazidime or ceftazidime-avibactam are used to treat the infection.

## References

[1] Bebrone C 2007 Metallo-β-lactamases (classification, activity, genetic organization, structure, zinc coordination) and their superfamily. Biochem Pharmacol 74:1686–1701. doi:10.1016/j.bcp.2007.05.021. 17597585

[2] Maltezou HC 2009 Metallo-β-lactamases in Gram-negative bacteria: introducing the era of pan-resistance? Int J Antimicrob Agents 33:405.e1–405.e7. 19095416 10.1016/j.ijantimicag.2008.09.003

[3] Walsh TR 2005 The emergence and implications of metallo-β-lactamases in Gram-negative bacteria. Clin Microbiol Infect 11(Suppl):2–9. doi:10.1111/j.1469-0691.2005.01264.x. 16209700

[4] Khosravi Y, Tay ST, Vadivelu J 2011 Analysis of integrons and associated gene cassettes of metallo-β-lactamase-positive Pseudomonas aeruginosa in Malaysia. J Med Microbiol 60:988–994. doi:10.1099/jmm.0.029868-0. 21436370

[5] Walsh TR, Toleman MA, Poirel L, Nordmann P 2005 Metallo-β-lactamases: the quiet before the storm? Clin Microbiol Rev 18:306–325. doi:10.1128/CMR.18.2.306-325.2005. 15831827 PMC1082798

[6] Ramírez MS, Pineiro S, Argentinian Integron Study Group, Centron D. 2010 Novel insights about class 2 integrons from experimental and genomic epidemiology. Antimicrob Agents Chemother 54:699–706. doi:10.1128/AAC.01392-08. 19917745 PMC2812161

[7] Sabbuba NA, Mahenthiralingam E, Stickler DJ 2003 Molecular epidemiology of Proteus mirabilis infections of the catheterized urinary tract. J Clin Microbiol 4:4961–4965. 14605124 10.1128/JCM.41.11.4961-4965.2003 PMC262497

[8] Fowler RC, Hanson ND 2014 Emergence of carbapenem resistance due to the novel insertion sequence ISPa8 in Pseudomonas aeruginosa. PLoS One 9(3):e91299. doi:10.1371/journal.pone.0091299. 24614163 PMC3948848

[9] Moland ES, Kim S-Y, Hong SG, Thomson KS 2008 Newer β-lactamases: clinical and laboratory implications, part II. Clin Microbiol Newsl 30:79–85. doi:10.1016/j.clinmicnews.2008.05.001.

[10] Clinical and Laboratory Standards Institute. 2012 Performance standards for antimicrobial susceptibility testing; 22nd informational supplement. CLSI M100-S22. Clinical and Laboratory Standards Institute, Wayne, PA.

[11] Tada T, Nhung PH, Miyoshi-Akiyama T, Shimada K, Phuong DM, Anh NQ, Qhmagari N, Kirikae T 2015 IMP-51, a novel IMP-type metallo-β-lactamase with increased doripenem- and meropenem-hydrolyzing activities, in a carbapenem-resistant Pseudomonas aeruginosa clinical isolate. Antimicrob Agents Chemother 59:7090–7093. doi:10.1128/AAC.01611-15. 26282421 PMC4604376

[12] Sanders CC, Sanders WE Jr, Moland ES 1986 Characterization of β-lactamases *in situ* on polyacrylamide gels. Antimicrob Agents Chemother 30:951–952. doi:10.1128/AAC.30.6.951. 3492960 PMC180628

[13] Roth AL, Hanson ND 2013 Rapid detection and statistical differentiation of KPC gene variants in Gram-negative pathogens by use of high-resolution melting and ScreenClust analyses. J Clin Microbiol 51:61–65. doi:10.1128/JCM.02193-12. 23077125 PMC3536237

[14] Barlow RS, Gobius KS 2006 Diverse class 2 integrons in bacteria from beef cattle sources. J Antimicrob Chemother 58:1133–1138. doi:10.1093/jac/dkl423. 17065187

[15] Hansson K, Sundstrom L, Pelletier A, Roy PH 2002 IntI2 integron integrase in Tn*7*. J Bacteriol 184:1712–1721. doi:10.1128/JB.184.6.1712-1721.2002. 11872723 PMC134885

[16] Wei Q, Hu Q, Li S, Lu H, Chen G, Shen B, Zhang P, Zhou Y 2014 A novel functional class 2 integron in clinical Proteus mirabilis isolates. J Antimicrob Chemother 69:973–976. doi:10.1093/jac/dkt456. 24235093

[17] Partridge SR, Tsafnat G, Coiera E, Iredell JR 2009 Gene cassettes and cassette arrays in mobile resistance integrons. FEMS Microbiol Rev 33:757–784. doi:10.1111/j.1574-6976.2009.00175.x. 19416365

[18] Collis CM, Hall RM 1995 Expression of antibiotic resistance genes in the integrated cassettes of integrons. Antimicrob Agents Chemother 39:155–162. doi:10.1128/AAC.39.1.155. 7695299 PMC162502

[19] da Fonseca EL, dos Santos Freitas F, Vicente AC 2011 Pc promoter from class 2 integrons and the cassette transcription pattern it evokes. J Antimicrob Chemother 66:797–801. doi:10.1093/jac/dkr011. 21393219

[20] Gillings MR 2014 Integrons: past, present, and future. Microbiol Mol Biol Rev 78:257–277. doi:10.1128/MMBR.00056-13. 24847022 PMC4054258

[21] Cambray G, Guerout A, Mazel D 2010 Integrons. Annu Rev Genet 44:141–166. doi:10.1146/annurev-genet-102209-163504. 20707672

[22] Stokes HW, O'Gorman DB, Recchia GD, Parsekhlan M, Hall RM 1997 Structure and function of 59-base element recombination sites associated with mobile gene cassettes. Mol Microbiol 26:731–745. doi:10.1046/j.1365-2958.1997.6091980.x. 9427403

[23] Hall RM 2012 Integrons and gene cassettes: hotspots of diversity in bacterial genomes. Ann N Y Acad Sci 1267:71–78. doi:10.1111/j.1749-6632.2012.06588.x. 22954219

[24] Partridge SR 2011 Analysis of antibiotic resistance regions in gram-negative bacteria. FEMS Microbiol Rev 35:820–855. doi:10.1111/j.1574-6976.2011.00277.x. 21564142

[25] Recchia G, Hall RM 1995 Gene cassettes: a new class of mobile element. Microbiology 141:3015–3027. doi:10.1099/13500872-141-12-3015. 8574395

[26] Stokes HW, Hall RM 1989 A novel family of potentially mobile DNA elements encoding site-specific gene-integration function: integrons. Mol Microbiol 3:1669–1683. doi:10.1111/j.1365-2958.1989.tb00153.x. 2560119

[27] Hall RM, Brookes DE, Stokes HW 1991 Site-specific insertion of genes into integrons: role of the 59-base element and determination of the recombination cross-over point. Mol Microbiol 5:1941–1959. doi:10.1111/j.1365-2958.1991.tb00817.x. 1662753

[28] Johansson C, Kamali-Moghaddam M, Sundstrom L 2004 Integron integrase binds to bulged hairpin DNA. Nucleic Acids Res 32:4033–4043. doi:10.1093/nar/gkh730. 15289577 PMC506814

[29] Mazel D 2006 Integrons: agents of bacterial evolution. Nat Rev Microbiol 4:608–620. doi:10.1038/nrmicro1462. 16845431

[30] Tijet N, Lo S, Siebert H, MacNeill M, Rawte P, Farrell DJ, Low DE, Patel SN, Melano RG 2012 Detection of IMP-27 metallo-β-lactamase in Proteus mirabilis, ON, Canada, abstr C2-090 Abstr 52nd Intersci Conf Antimicrob Agents Chemother.

